# Characterization of Free and Glycosidically Bound Volatile and Non-Volatile Components of Shiikuwasha (*Citrus depressa* Hayata) Fruit

**DOI:** 10.3390/foods13213428

**Published:** 2024-10-28

**Authors:** Aldia Katherinatama, Yonathan Asikin, Kazuki Shimoda, Momoko Shimomura, Fumimasa Mitsube, Kensaku Takara, Koji Wada

**Affiliations:** 1 Department of Bioscience and Biotechnology, Faculty of Agriculture, University of the Ryukyus, 1 Senbaru, Nishihara 903-0213, Okinawa, Japan; 2 United Graduate School of Agricultural Sciences, Kagoshima University, 1-21-24 Korimoto, Kagoshima 890-0065, Kagoshima, Japan; 3 Okinawa Prefectural Agricultural Research Center Nago Branch, 4605-3, Nago 905-0012, Okinawa, Japan; 4 Hokubu Agriculture, Forestry and Fisheries Promotion Center, Okinawa Prefectural Government, 1-13-11 Ominami, Nago 905-0015, Okinawa, Japan

**Keywords:** Shiikuwasha, cultivation line, free volatile, released volatile, non-volatile compound

## Abstract

Shiikuwasha, a citrus fruit native to Okinawa, Japan, has various cultivation lines with distinct free volatile and non-volatile components. However, the glycosylated volatiles, which are sources of hidden aromas, remain unknown. This study aimed to characterize the chemical profiles of free and glycosidically bound volatile as well as non-volatile components in the mature fruits of six Shiikuwasha cultivation lines: Ishikunibu, Izumi kugani-like, Kaachi, Kohama, Nakamoto seedless, and Ogimi kugani. Free volatiles were analyzed using solid-phase microextraction–gas chromatography–mass spectrometry. Glycosides were collected via solid-phase extraction and hydrolyzed with *β*-glucosidase, and the released volatiles were measured. Additionally, the non-volatile components were determined using non-targeted proton nuclear magnetic resonance analysis. Total free and bound volatiles ranged from 457 to 8401 µg/L and from 104 to 548 µg/L, respectively, and the predominant free volatiles found were limonene, *γ*-terpinene, and *p*-cymene. Twenty volatiles were released from glycosides, including predominant 1-hexanol and benzyl alcohol, with Kaachi and Ogimi kugani showing higher concentrations. Principal component analysis (PCA) revealed that taste-related compounds like sucrose, citrate, and malate influenced line differentiation. The PCA of the combined data of free and bound volatile and non-volatile components showed flavor component variances across all lines. These findings provide valuable insights into the chemical profiles of Shiikuwasha fruits for fresh consumption and food and beverage processing.

## 1. Introduction

Shiikuwasha (*Citrus depressa* Hayata) is a typical small citrus fruit with a highly sour flavor and strong distinctive aroma. It is grown in the northern portion of Okinawa Island and is predominantly planted at subtropical temperatures, requiring well-drained soil and abundant sunlight [1]. The Okinawan Shiikuwasha plant grows in various cultivation lines and produces approximately 3000 tons of fresh fruit, juice, and food additives per year [2]. Shiikuwasha is well known for its polymethoxyflavone components nobiletin and tangeretin, which have a wide range of biofunctions, including anti-inflammatory, anticancer, antidiabetic, and anti-osteoarthritis properties [3,4]. It is also known for possessing a distinct volatile profile with less limonene and more *γ*-terpinene than other Japanese citrus varieties [3]. These volatiles have anti-inflammatory, antioxidant, and antibacterial properties [5].

The aroma characteristics of citrus, which are affected by volatile organic compounds, are important attributes that distinguish citrus varieties, each with its own flavor [6]. These compounds, which are mostly found in free forms, include monoterpenes, sesquiterpenes, esters, aldehydes, ketones, and alcohols, and thus profoundly influence the fruit and its derived products [7]. Volatile hydroxy compounds can also be stored in the plants as glycosylated forms that are bonded to a sugar moiety [8]. Henceforth, the UDP-glucosyltransferase (UGT) enzyme catalyzes the attachment of a sugar moiety to those compounds, enhancing their stability and enabling controlled release in plants [8]. Upon hydrolysis, these compounds can be liberated in their volatile form, thus regaining their odor [9]. The chemical composition of glycoside compounds can be analyzed using liquid chromatography–mass spectrometry (LC-MS); however, the glycosides could be hydrolyzed using *β*-glucosidase, and the release of volatiles over enzymatic hydrolysis could be determined using gas chromatography–mass spectrometry (GC-MS) [10]. This approach is straightforward yet effective for analyzing glycosylated compounds in fruits. Understanding the composition of free volatiles in citrus can help to improve the sensory aspects of food products, whereas studying glycosidically bound volatiles is crucial for increasing the overall flavor quality of citrus products.

Taste, which is one of the most essential aspects of fruit quality, is primarily determined by sugar levels and organic acid ratios, and is influenced by their components [11]. These components play a key role in both primary metabolism in fruits and the production of various metabolites, such as sugars, amino acids, organic acids, and vitamins, all of which influence fruit quality [12]. The combination of sugars, amino acids, organic acids, and vitamins in citrus fruits creates a complex and dynamic flavor profile that is essential for their sensory appeal. The balance between sweetness and tartness, along with subtle flavor nuances from amino acids and the health-associated benefits of vitamins, make citrus fruits highly desirable. Chromatography, which comprises liquid or gas separation phases, is a typical technique for determining the composition of foods, and nuclear magnetic resonance (NMR) may also be used as an alternative approach to analyze non-volatile components [13]. Each technique has its own advantages and limitations, including speed, selectivity, dynamic range, and ease of use. For example, NMR has a lower sensitivity than mass spectrometry (MS), but it offers a more consistent metabolite structure and does not require destructive sample preparation [14]. Moreover, non-targeted NMR is a useful tool for examining the chemical composition and molecular dynamics of food matrices, as it allows for the non-invasive examination of complicated compositions in solid, liquid, and semisolid forms [14,15].

The cultivation line is one of the essential factors for differentiating free volatile composition of Okinawan Shiikuwasha [16]. However, to the best of our knowledge, there have been no investigations of glycosidically bound volatile compounds in Okinawan Shiikuwasha fruit. Moreover, there is a lack of information regarding the use of NMR techniques to determine the non-volatile components in Shiikuwasha. Although glycosidically bound volatiles are not volatiles, their releases during food processing might add to the aroma of the foods [17]. Non-volatile components such as sugars and organic acids also affect the perceived flavor properties of the foods [17,18]. This study of these substances along with free volatile components offers a thorough understanding of the potential industrial applications of local fruits, including Shiikuwasha, in the food applications.

Therefore, this study aimed to characterize the free and glycosidically bound volatile and non-volatile components of Shiikuwasha fruits in six major cultivation lines: Ishikunibu, Izumi kugani-like, Kaachi, Kohama, Nakamoto seedless, and Ogimi kugani. This is the first report on compounds released from glycosidically bound volatile components in Okinawan Shiikuwasha fruit and multivariate analysis of the combined data of free and glycosidically bound volatile and non-volatile components.

## 2. Materials and Methods

### 2.1. Chemicals and Reagents

Deuterium oxide (D_2_O), 2,2-dimethyl-2-silapentane-5-sulfonate (DSS) sodium salt, and 1-hexanol (99.5% purity) were purchased from Sigma-Aldrich (St. Louis, MO, USA). Cyclohexanol (98% purity) was purchased from Fujifilm Wako Pure Chemical Corporation (Osaka, Japan). Rapidase Revelation Aroma was obtained from DSM Food Specialties (Delft, The Netherlands). All other reagents were analytical-grade.

### 2.2. Shiikuwasha Juice Preparation

Mature Shiikuwasha fruits from six cultivation lines (Ishikunibu, Izumi kugani-like, Kaachi, Kohama, Nakamoto seedless, and Ogimi kugani) were harvested at their mature stage in December 2021 from a farm at the Okinawa Prefectural Agricultural Research Center’s Nago branch. The weight and total soluble solids of the mature fruits ranged from 33.30 to 47.60 g and from 8.43 to 10.33 °Brix, respectively (Supplementary Table S1). The fruits (2–2.5 kg) from the outer sections of the plants were mixed and then equally divided into three groups. The fruits of each batch were peeled, and their edible parts were separately squished using a slow juicer ISJ-56-W (Iris Ohyama, Sendai, Miyagi, Japan) in an air-conditioned room (25 °C) (experimental replication was carried out on the juice extraction process). The resulting juice was then stored in a sealed vial at −30 °C prior to analysis.

### 2.3. Free Volatile Compound Analysis

The free volatile components of Shiikuwasha were extracted using an SPME fiber coated with divinylbenzene/carboxen/polydimethylsiloxane 50/30 µm (Supelco, Bellefonte, PA, USA) and evaluated using GC-MS (Agilent Technologies, Santa Clara, CA, USA) equipped with a CombiPAL autosampler (CTC Analytics, Zwingen, Switzerland). Briefly, 2 mL of Shiikuwasha juice, 10 µL of internal standard 1-hexanol (0.5 mg/mL in H_2_O), and 1 mL of EDTA (0.15 M, pH 7.5) were taken in a 20 mL vial and mixed at 40 °C for 5 min. The addition of EDTA solution was used to inhibit any enzymatic reaction that could have occurred prior to GC-MS analysis. A preconditioned SPME fiber was then introduced, and the volatiles in the headspace were extracted at 40 °C for 10 min. GC-MS analysis was performed using a 7890 B GC-5977A (Agilent Technologies). The column employed was DB5-MS or DB-Wax (30 m × 0.25 mm, 0.25 μm), with an inlet temperature of 250 °C and a split ratio of 10:1, and helium was used as the carrier gas at a constant flow rate of 1 mL/min. The oven temperature program was set at 50 °C at a rate of 4 °C/min, increase to 150 °C at 10 °C/min, and finally held at 200 °C for 6.25 min. MS acquisition and ionization were performed for *m*/*z* 33–450 in electron ionization mode (70 eV). The ion source and transfer line temperatures were both set to 230 °C. Volatile compounds were identified based on their MS similarities to the MS data obtained from the NIST 17 library (>80%) and linear retention index (RI) comparisons (<|20|). The weight intensity of the peaks was calibrated to the internal standard’s response, and volatile compounds were represented in µg/L of juice. All assays were performed in triplicates.

### 2.4. Glycosidically Bound Volatile Compound Analysis

The glycosidically bound volatile components of Shiikuwasha were extracted using solid-phase extraction. Briefly, Shiikuwasha juice (500 mL) was centrifuged at 10,000× *g* for 20 min at 20 °C (Hitachi CR22GIII, Eppendorf Himac Technologies, Ibaraki, Japan), and the resulting supernatant (150 mL) was loaded into an open column (50 × 1 cm) containing 50 g of Diaon HP-20 resin (Sigma-Aldrich). Juice was retained in the column for 1 min and passed through the column at a flow rate of 0.5 mL/min. Next, 100 mL of distilled water was eluted three times. Subsequently, 80 mL of pentane:dichloromethane (1:1, *v*/*v*) was eluted three times to remove lipids and free volatile compounds. The glycoside fraction was extracted using 80 mL of 100% methanol at a flow rate of 0.5 mL/min. The resulting fraction was concentrated using a vacuum evaporator (Eyela N-1300; Eyela, Tokyo, Japan). The dried glycoside extract was then dissolved in 2 mL of 0.2 M citric buffer (pH 4.5) and 200 µL of 20 mg/mL Rapidase Revelation Aroma solution (an enzyme premix from *Aspergillus niger* containing *β*-glucosidase, enzymatic activity > 4000 u/g) in 0.2 M citric buffer. The mixture was incubated at 40 °C for 2 h. Then, 500 μL of 20% NaCl solution was added to inhibit any further hydrolysis reaction prior to analysis. The liberated volatiles were analyzed using SPME-GC-MS via an internal standard addition (20 μL cyclohexanol, 0.1 mg/mL in H_2_O). A reaction without *β*-glucosidase was used as a negative control. SPME-GC-MS analysis was conducted for free volatile measurements. The released compounds were measured from the hydrolyzed extract and represented in µg/L of juice. All assays were performed in triplicates.

### 2.5. Non-Volatile Component Analysis

The non-volatile components of Shiikuwasha were examined using ^1^H-NMR. Briefly, 140 µL of Shiikuwasha juice and 560 µL of D_2_O solution containing 1 mM DSS (internal standard in 500 mM phosphate buffer, pH 7.0) were placed into a 2 mL microtube. The mixture was then transferred to a 5 mm NMR tube and analyzed using ^1^H-NMR at Bruker AVANCE-500 spectrometer (Bruker, Karlsruhe, Germany). DSS was utilized for chemical shift reference (*δ* = 0 ppm) and as an internal standard for quantitative analysis. The NMR parameters were set at a frequency of 500.23 MHz, a ZGPR pulse program, 64 times the number of measurements, and a 4 s mitigation time. The data were identified and quantified using the Chenomx NMRSuite (Version 10.1, Chenomx, Edmonton, AB, Canada). All assays were performed in triplicates.

### 2.6. Statistical Analysis

The Tukey–Kramer significant difference test (JMP, SAS Institute, Cary, NC, USA) was used to assess differences between samples. Principal component analysis (PCA), PLS-DA (partial least squares discriminant analysis), and HCA (hierarchical cluster analysis) multivariate plots were constructed using SIMCA Version 17 (Sartorius, Göttingen, Germany).

## 3. Results

### 3.1. Total Content of Free and Glycosidically Bound Volatiles of Shiikuwasha Fruit

**Figure 1:**
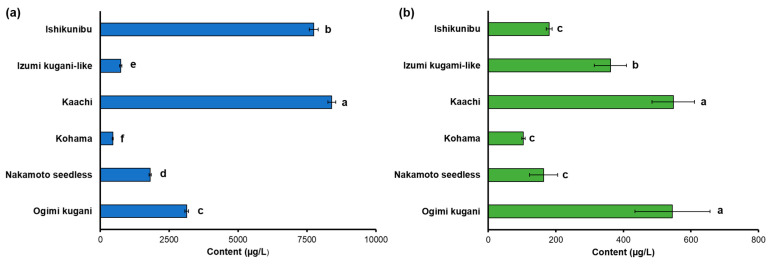
Total volatile content of Shiikuwasha fruit of different cultivation lines. (**a**) Free volatiles and (**b**) glycosidically bound volatiles. Each value is expressed as the mean ± standard deviation of three replicates. Means followed by the same letter are not significantly different at *p* < 0.05; *n* = 3.

Free and glycosidically bound volatiles are two essential aroma compounds that contribute to the flavor characteristics of Shiikuwasha fruit. The total content of free and glycosidically bound volatiles of the Shiikuwasha of different cultivation lines ranged from 457 to 8401 and from 103.89 to 547.65 µg/L, respectively (Figure 1). The Kaachi line contained the highest amount of total free volatiles, followed by Ishikunibu (8401 vs. 7744 µg/L, respectively) (Figure 1a). Moreover, the total free volatiles varied significantly among other lines, in which Ogimi kugani, Nakamoto seedless, Izumi kugani-like, and Kohama accounted for 3135, 815, 743, and 456 µg/L, respectively (*p* < 0.05). On the other hand, the Kaachi and Ogimi kugani lines had significantly greater amounts of released volatiles from their glycoside fractions, which accounted for 547.65 and 545.31 µg/L, respectively, followed by Izumi kugani-like (361.53 µg/L) (Figure 1b). The other lines contained fewer total bound volatiles in the range of 103.89 to 180.09 µg/L.

### 3.2. Free Volatile Components of Shiikuwasha Fruit

A total of 37 free volatile components were detected in six cultivation lines of Shiikuwasha fruit, comprising 15 monoterpene hydrocarbons, six monoterpene alcohols, five sesquiterpenes, five aldehydes, two monoterpene ketones, one monoterpene ether, one alcohol, one ester, and one ether (Table 1). The predominant free volatiles of these cultivation lines were limonene, *γ*-terpinene, and *p*-cymene, which were in the ranges of 251.39–5649.59, 23.56–1559.85, and 59.05–976.78 µg/L, respectively. The free volatiles between the cultivation lines therefore varied significantly (*p* < 0.05). Moreover, dehydrosabinene, (*E*)-carveol, and citronellol were only detected in the Ishikunibu line, whilst 1,8-cineole and α-bergamotene were solely found in Kohama and Kaachi, respectively. On the other hand, linalool was discovered to be the most aroma-active compound, with the Kaachi and Ishikunibu lines having the highest odor activity values (OAVs), 282.60 and 230.13, respectively (Table 2). In addition, the Shiikuwasha juice of different cultivation lines contained seven more aroma-active compounds of free volatiles, including limonene, *p*-cymene, and octanal, with OAVs in the ranges of 7.39–166.16, 3.77–73.44, and 4.53–57.28, respectively.

**Figure 2:**
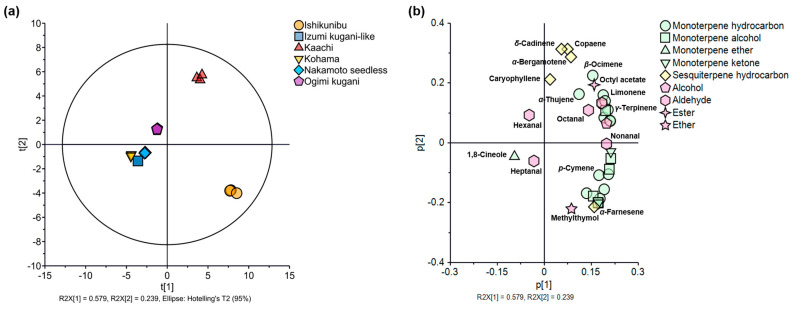
(**a**) PCA score plots and (**b**) factor loadings of free volatile compounds of Shiikuwasha fruit of different cultivation lines.

The variations in free volatile components between cultivation lines were further explained by multivariate analysis using PCA. The first two principal components (PCs) of the free volatile compounds of Shiikuwasha from the different cultivation lines explained 81.8% of the total variance, accounted for by PC1 and PC2 (57.9 and 23.9%, respectively) (Figure 2). The score plots of each line were outlined close to one another, indicating that the replicates had comparable volatile compound profiles (Figure 2a). The PCA score plots showed variations among all cultivation lines based on the relative concentrations (normalized intensity) of their free volatiles, in which two lines (Kaachi and Ishikunibu) were distinctively separated from the other plots. The Kaachi line was positive for both PC1 and PC2, whereas Ishikunibu was plotted in the positive direction of PC1 and negative direction for PC2. Moreover, Ogimi Kugani was plotted near the zero coordinate of both PCs, but it was in the negative direction of PC1 and the positive direction of PC2. The other three lines (Izumi kugani-like, Kohama, and Nakamoto seedless) were plotted in a negative direction for both PCs and were close to one another. Factor loading plots were used to investigate the relationships between the score plots and respective free volatile compounds. Most free volatiles were outlined in the positive direction of PC1 (Figure 2b), including monoterpenes (monoterpene hydrocarbons, monoterpene alcohols, and monoterpene ketones) and sesquiterpene hydrocarbons. Moreover, alcohol and ester compounds were placed on the positive sides of both PCs. Additionally, methylthymol was plotted in the positive direction of PC1 and negative direction of PC2, whereas hexanal, heptanal, and 1,8-cineole were outlined in the negative plot of PC1. The Kaachi and Ishikunibu lines had higher relative concentrations of monoterpenes and sesquiterpenes than the other lines; therefore, they could be distinguished by the concentration of total terpenes, which were the most prevalent volatile classes in both fruits (Figure 2; Table 1). The Kaachi score plots were influenced by the intensities of monoterpene hydrocarbons (limonene, *γ*-terpinene, *α*-thujene, and *β*-ocimene), sesquiterpene hydrocarbons (copaene, *δ*-cadinene, *α*-bergamotene, and caryophyllene), octanal, and octyl acetate.

### 3.3. Glycosidically Bound Volatile Components of Shiikuwasha Fruit

Twenty volatile compounds were liberated from their glycosidic forms via enzymatic hydrolysis, comprising 12 aliphatic alcohols, four aromatic alcohols, and four monoterpene alcohols (Table 3). The Kaachi line contained 18 glycosylated volatiles, while the number of volatiles released from the glycosides ranged from 11 to 15 in the other cultivation lines. Notably, the Kaachi and Ogimi kugani lines had significantly greater amount of 1-hexanol (284.01 and 212.62 µg/L, respectively) (*p* < 0.05). These two lines contained moderate amounts of benzyl alcohol (70.20 and 145.29 µg/L, respectively). In contrast, the most prevalent compound in the other cultivation lines was benzyl alcohol, ranging from 26.79 to 83.38 µg/L. 3-Octanol was found in the Kaachi and Ogimi kugani lines, whereas 2-octanol was found only in Kaachi. Moreover, phenylethyl alcohol was released from glycosides derived from all cultivation lines from 1.82 to 14.05 µg/L, except for Kohama. Methyl salicylate was found in all lines, with the Izumi kugani-like line showing the highest content (68.83 µg/L). Thymol was also found only in two cultivation lines, i.e., Izumi kugani-like (17.65 µg/L) and Kohama (2.76 µg/L).

**Figure 3:**
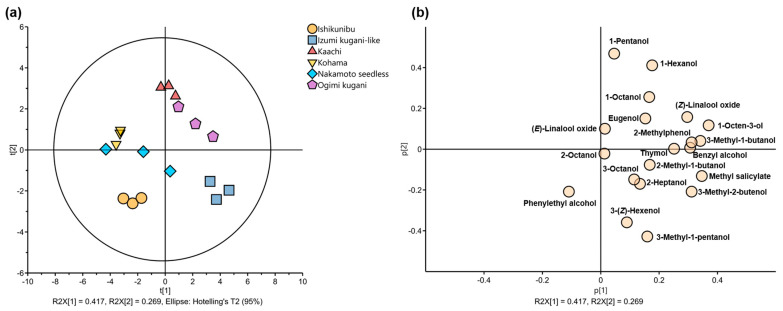
(**a**) PCA score plots and (**b**) factor loadings of released compounds from glycosidically bound volatile compounds of Shiikuwasha fruit of different cultivation lines.

The PCA plots further displayed the first two PCs that explained 41.7 and 26.9% of the variance in the glycosylated volatiles (Figure 3). Despite the dispersed score plots of some cultivation lines, e.g., Nakamoto seedless and Ogimi kugani, the plots were still reasonably close to one another, making it feasible to identify the distinctive bound volatile characteristics of each line (Figure 3a). The compounds were distributed in three quadrants of the factor loading plot, except in the negative direction of PC1 and positive direction of PC2 (Figure 3b), which showed that the Kohama line lacked glycosylated volatile content compared to others. On the other hand, the Kaachi and Ogimi kugani lines, which were plotted not very distant from each other at the upper quadrant of PC1, could be influenced by 1-hexanol and 1-pentanol loadings. Moreover, the separation of the Izumi kugani-like and Ishikunibu lines in the negative direction of PC2 could be affected by phenylethyl alcohol, 3-(*Z*)-hexenol, and 3-methyl-1-pentanol.

### 3.4. Non-Volatile Components of Shiikuwasha Fruit

**Figure 4:**
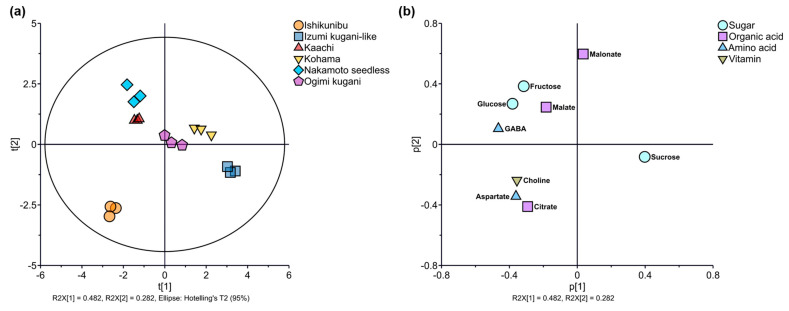
(**a**) PCA score plots and (**b**) factor loadings of non-volatile components of Shiikuwasha fruit of different cultivation lines.

Three sugars, four organic acids, one amino acid, and one vitamin were detected in Shiikuwasha fruit (Table 4). The Kohama and Izumi Kugani-like lines contained significantly greater sucrose (36.21 and 33.38 mM, respectively) than other cultivation lines (*p* < 0.05). Moreover, the most prevalent non-volatile components detected in the Kaachi and Nakamoto seedless lines were fructose, glucose, and GABA. On the other hand, the Kaachi, Ogimi kugani, and Kohama lines had significantly higher malate content (4.80, 4.78, and 4.72 mM, respectively). Moreover, Nakamoto seedless possessed the highest malonate content compared to other cultivation lines (0.65 mM). In contrast, citrate, aspartate, and choline were the most-detected non-volatiles in Ishikunibu (42.04, 2.04, and 0.15 mM, respectively). Accordingly, the first two PCs further explained 76.4% of the variance and were accounted for by PC1 and PC2 (48.2% and 28.2%, respectively) (Figure 4). The score plots of each cultivation line were outlined separately, demonstrating that each line had distinct non-volatile components (Figure 4a). The scores of the Ogimi kugani line were plotted near the zero coordinates of both PCs. The Kohama line was plotted in the positive direction for both PCs, whereas the Nakamoto and Kaachi lines were negative for PC1 and positive for PC2. The Izumi kugani-like line was plotted in the positive direction of PC1 and negative direction of PC2. In addition, the Ishikunibu line was outlined in the negative direction for both PCs. Most of the factor loadings of non-volatile components, which comprised taste-related compounds, such as sugars, organic acids, and nutrient substances (amino acids and vitamins), were outlined in the negative direction of PC1 (Figure 4b). They were fructose, glucose, malate (malic acid), and GABA in the positive direction of PC2, and citrate (citric acid), aspartate (aspartic acid), and choline in the negative direction of PC2. Conversely, sucrose was distinctively separated in the positive direction of PC1 and negative direction of PC2, whereas malonate (malonic acid) was plotted near the zero coordinate of PC1 but in the positive direction of PC2.

### 3.5. Multivariate Statistical Plots of the Combined Data of Free and Glycosidically Bound Volatile and Non-Volatile Components of Shiikuwasha Fruit

**Figure 5:**
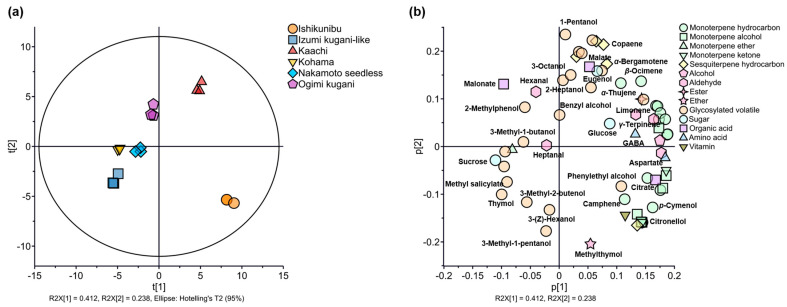
(**a**) PCA score plots and (**b**) factor loadings of the combined normalized intensity data of free and glycosidically bound volatile and non-volatile components of Shiikuwasha fruit of different cultivation lines.

The effects of free volatiles, glycosylated volatiles, and non-volatile components on the Shiikuwasha differentiation of various cultivation lines were further investigated using PCA, PLS-DA, and HCA (Figure 5, Supplementary Figure S1). All six cultivation lines are outlined separately in the score plots of the combined data, showing an improved multivariate distribution pattern compared with the PCA score plots of free volatiles only (Figure 5a vs. Figure 5b). The Kaachi and Ishikunibu lines were in the positive directions of both PCs, while the Ogimi kugani line was plotted near the zero coordinate of PC1, but in the positive direction of PC2. Interestingly, the three cultivation lines (Kohama, Nakamoto seedless, and Izumi kugani-like) that could not be separated by PCA-free volatiles were completely detached from one another in the negative direction of both PCs. The PCA factor loadings of these combined data consisted of most of the free volatiles in the positive direction for both PCs, except for hexanal and heptanal (Figure 5b). These data confirmed the dominance of the Kaachi and Ishikunibu cultivation lines in total volatile content (Figure 1). Moreover, although glycosylated bound volatiles were drawn in all quadrants of the loading plots, eight released volatiles, including methyl salicylate and thymol, profoundly contributed to the distinctiveness of the Kohama, Nakamoto seedless, and Izumi kugani-like lines. The differentiation of these lines could also be augmented by their sucrose levels, as plotted in the negative direction for both the PCs. Additionally, the loading plot of citrate might also contribute to the overall differentiation of all cultivation lines as a distinct separation of the Ishikunibu line in the positive direction of PC1 and negative direction of PC2.

The differentiation in Shiikuwasha cultivation lines was validated using PLS-DA, wherein similar score plots were also obtained by PLS-DA as compared to PCA score plots, suggesting that the main sources of variation are identical for both calculations (Figure 5a; Supplementary Figure S1a). The PLS-DA calculation also provided the importance of different compounds in discriminating between cultivation lines via variable importance in projection (VIP) values (Supplementary Table S2). The top ten discriminatory substances included four free volatiles (heptanal, hexanal, eugenol, and 1,8-cineole), two sugars (sucrose and glucose), and four glycosidically bound volatiles (3-*(Z)*-hexenol, 3-methyl-1-butanol, 3-methyl-2-butenol, and 2-methyl-1-butanol) (Supplementary Table S2). The HCA visualization plot was used to further confirm the classification of cultivation lines (Supplementary Figure S1c). It showed an isolated branch of the Ishikunibu line, with Kaachi and Ogimi kugani clustered close to it. The other three lines (Izumi kugani-like, Kohama, and Nakamoto seedless) were separated into different branches.

## 4. Discussion

The total content of free and glycosidically bound volatiles varies among Shiiku-washa cultivation lines due to differences in released volatiles (Figure 1). This variation highlights the preservation of the distinct traits in each line, attributed to genetic factors. Genetic polymorphisms influence the synthesis and metabolism of volatile organic compounds, altering the accumulation of free and glycosidically bound volatiles. Free volatiles in citrus, predominantly synthesized through the mevalonic acid (MVA) and methylerythritol phosphate (MEP) pathways, undergo enzymatic conversion into various compounds, influenced by terpene synthase (TPS) expression patterns in these pathways [20,21]. On the other hand, the UGT enzyme catalyzes the conjugation of sugar molecules, particularly glucose, to volatile compounds, forming glycosides [21]. In citrus fruits, many volatile compounds exist as non-volatile glycoconjugates, such as mono- or diglycosides linked to aglycones like terpenes and phenolics, known for enhancing fruit flavor [22]. It is therefore inferred that cultivated lines such as Kaachi might show an elevated expression of TPS and UGT enzymes, leading to enhanced concentrations of terpenes and bound volatiles, respectively.

In citrus fruits, free volatiles are typically more abundant than glycosidically bound volatiles [23]. Free volatiles can easily evaporate and migrate from the fruit, directly enhancing the aroma and presenting various fragrances; however, glycosidically bound volatiles must be cleaved from their sugar molecules before contributing to the aroma [24]. This indicates that the release of glycosidically bound volatiles requires specific conditions or actions where aroma aglycones can be liberated during fruit ripening, postharvest storage, and acidic or enzymatic hydrolysis, which may not always occur during typical consumption [25]. Thus, these free and bound volatiles are major contributors to the aroma quality of citrus fruits, including that of Shiikuwasha, and they impart a characteristic aroma that is perceived by the olfactory and gustatory senses and their sensory attributes [26,27]. The total free and bound volatiles in Shiikuwasha from various cultivation lines may influence their aroma properties and potent practical food applications when fruit-derived products are processed using enzymatic hydrolysis.

The free volatile data can be visualized using multivariate statistical analysis, such as PCA. The primary application of PCA is to represent a multivariate data table as a smaller number of variables in order to identify trends, leaps, clusters, and outliers [28]. The PCA plot can aid in the removal of noise and redundant features from data by focusing on the main components that capture the greatest variance [29]. Thoroughly, octanal may contribute to fruity, oily, and woody aromas in the Kaachi line [30]. In contrast, Ishikunibu, which was plotted at positive to PC1 and negative to PC2, was influenced by *p*-cymene, *α*-farnesene, and methylthymol (Figure 2). Therefore, citrus-like and herbaceous aromas can be augmented in Kaachi and Ishikunibu fruits by monoterpenes and sesquiterpenes, respectively [31,32]. In addition, Ogimi kugani could be distinguished from other lines by its distinct green-grassy odor from hexanal, which may be used as a possible chemical marker of the fruit [33]. The distinct compounds in the Izumi kugani-like, Kohama, and Nakamoto seedless lines were 1,8-cineole (minty-herbal) and heptanal (fruity-green) [32,33]. Detailed aroma compound data characterized by monoterpenes, sesquiterpenes, aldehydes, and other volatiles were revealed by analyzing the free volatile components in Shiikuwasha across six cultivation lines (Table 1). These thorough data offer insightful information on the possible distinctive aroma qualities of these lines. The distinct and pleasant citrus scent is produced by monoterpene compounds such as limonene, linalool, and so on [34]. Eight free volatiles were identified as aroma-active components for having OAV greater than one (Table 2), indicating their major contribution to the odor strengths of Shiikuwasha [35]. In addition, the superior OAVs of limonene and linalool, which contribute fresh, citrusy, and floral notes, suggested that these key odor-contributing compounds dominated the overall aroma of the fruits [34]. The influence of the free volatile profiles on Shiikuwasha fruits derived from various cultivation lines could extend their use to a wide range of food applications. Understanding and leveraging these differences can drive innovation and enhance the sensory appeal and market competitiveness of Shiikuwasha cultivated on different lines.

The glycosylated volatiles can provide hidden aroma resources for Shiikuwasha fruits and their derived products when the glycosylated compounds are liberated through enzymatic hydrolysis (Table 3). Every liberated bound volatile could have released its own typical aroma trait that contributed to the unique aroma of Shiikuwasha across cultivation lines. More compounds were exposed in the factor loading plot of the positive direction of PC1, suggesting that fruits with high PC scores in the same direction were likely to contain higher quantities of these glycosylated volatiles, which in turn affected the fruits’ overall potential for concealed aromas (Figure 3). For instance, 1-hexanol, the most abundant glycosylated volatile, has a grassy and floral aroma, whereas benzyl alcohol has a pleasant flowery aroma when hydrolyzed from its bound forms [36]. Octanol can provide floral and fruity aromas, while methyl salicylate has a range of aromas, including mint, citrus, and woody [37]. Moreover, phenylethyl can contribute to citrus, floral, and sweet notes when volatilized [38]. Thymol is characterized by its herbaceous, spicy, and sometimes medicinal notes, thus contributing to the unique flavor profile of Shiikuwasha fruits and their processed products [39]. Although thymol only appeared in two cultivation lines and at relatively modest concentrations, it has gained substantial interest as an antibacterial agent, displaying strong antifungal activity and being an excellent dietary antioxidant [40]. Different citrus cultivation lines have distinct genetic backgrounds, resulting in variations in the enzymes responsible for synthesizing glycosidically bound volatiles. These enzymes play crucial roles in determining the types and quantities of volatile compounds produced by plants. Glycosidic precursors are composed of an aglycone (volatile compound) attached to a glycosidic unit (mono- or diglycoside) by a *β*-glycosidic linkage, resulting in non-volatile compounds with no odorous properties. These odorless sugar conjugates can be liberated during maturation, storage, and processing through acid or enzymatic hydrolysis, releasing free volatiles and possibly improving flavor quality [22]. The release of volatiles from glycosides adds complexity to the flavor profile of citrus products. Along with primary flavors, such as sweetness and acidity, the presence of secondary compounds enhances the overall sensory perception. This complexity is particularly important in products, such as citrus juices, for which consumers expect a well-balanced flavor profile. Therefore, careful consideration must be given to the use of released aglycones with strong odors in processed Shiikuwasha fruits, which may alter the overall flavor of the final products.

Non-volatile components that may affect the capacity to differentiate Shiikuwasha from various cultivation lines include taste-related substances like sucrose, citrate, and malate, as well as nutritional compounds like GABA and choline (Table 4; Figure 4). They are regarded as significant quality factors by both consumers and the food industry, as sugars are perceived to promote sweetness and organic acids promote sourness [41]. Citrus fruits are particularly rich in organic acids, and the acidic properties of organic acids are used, with sugar content as the key index of maturity and one of the major analytical measures of flavor quality [42]. Low concentrations of aspartic acid may not directly contribute to the taste of citrus; however, this amino acid may be involved in nitrogen metabolism and protein synthesis and serve as a precursor for the synthesis of various metabolites [43]. GABA and choline may be important additional sources of biofunction in Shiikuwasha lines in the negative direction of PC1 (Nakamoto seedless, Kaachi, and Ishikunibu) (Figure 4), with various potent health benefits, such as inhibitory neurotransmitter and cell membrane structure protection capabilities, respectively. Taken together, the intensity of taste compounds and nutritional substances varied in Shiikuwasha fruits depending on their cultivation lines. Thus, each fruit may exhibit variations in taste characteristics and provide different levels of bioactive substances, such as GABA and choline.

Multivariate analyses such as PCA, PLS-DA, and HCA of the combined data of free volatiles, glycosylated volatiles, and non-volatile components clearly demonstrated the uniqueness of flavor components in Shiikuwasha across different cultivation lines (Figure 5; Supplementary Figure S1). The combination of taste-related substances, particularly organic acids, can enhance or conceal specific aroma traits, thereby altering the overall aroma perception of Shiikuwasha fruit (Figure 5). Organic acids, such as citric and malic acids, contribute to the tartness and acidity of citrus fruits [43]. These acids can interact with volatile compounds to enhance aroma detection through chemical reactions, thereby enhancing the fruity and floral aroma perception of fruits. However, the effects of free and glycosidically bound volatiles, as well as other non-volatile components, on Shiikuwasha fruits of different cultivation lines and their derived products can be diverse and multifaceted. The differentiation of unseparated lines (Kohama, Nakamoto seedless, and Izumi kugani-like) in the PCA score of free volatiles could be clearly discriminated in the analysis of combined data (Figure 2 vs. Figure 5). Moreover, the loading score of the combined data showed the distribution of all compounds in the four quadrants of the plots, including glycosylated volatiles, indicating their contribution to the separation pattern. However, as glycosidically bound volatiles exhibited lower concentrations than free volatiles (Table 1 and Table 3), these glycosylated compounds did not extensively improve the discriminatory power between culture lines in the PCA score plots compared to free volatiles. Different citrus cultivation lines can exhibit variations in the composition and concentration of free and glycosidically bound volatiles, leading to diverse aroma profiles [22]. Moreover, PLS-DA calculation provided discriminant substances of free volatiles, glycosylated volatiles, and non-volatiles for VIPs greater than one (Supplementary Table S2). This highlights the significance of these three chemical categories in discriminating Shiikuwasha cultivation lines, and thus potently characterizes the overall flavor quality of the fruits and their derived processed foods. Taken together, this study provides information on volatile and non-volatile component profiles. These data can also be used to inform quality control, product development, and decision making in the citrus industry, ultimately improving the sensory quality and market competitiveness of Shiikuwasha derivatives. Farmers can use knowledge about the composition of fruits to select cultivation lines that are not only high-yielding, but also have desirable flavor profiles and nutritional content. Food manufacturers can utilize the diverse chemical composition of Shiikuwasha fruit to create unique and differentiated products with specific flavor profiles and nutritional benefits that appeal to niche markets and consumer preferences. Therefore, further investigation of the sensory profiles of the fruits and their derivatives is necessary, and the outcome of this current study can be used as basis data for further research such as sensomic comparison studies.

## 5. Conclusions

The six Shiikuwasha cultivation lines had different volatile profiles and non-volatile components, indicating that each cultivation was unique. The Kaachi line exhibited the highest total volatile content, suggesting that unique heredity influences volatile compound synthesis and metabolism, which potentially explains the variations in aroma profiles among all lines. The PCA score plots showed variations among all cultivation lines based on the relative concentrations of their free volatiles, with two lines, Kaachi and Ishikunibu, standing out from the others, with monoterpenes and sesquiterpenes being the most abundantly identified free volatiles. Twenty volatile compounds were released from their glycosidic forms upon enzymatic hydrolysis, and the Kaachi line produced 18 glycosylated volatiles. The Kaachi and Ogimi kugani lines had greater liberation of 1-hexanol, benzyl alcohol, 1-octanol, and 3-octanol. Except for the Kohama line, all lines released phenylethyl alcohol, with the Izumi Kugani-like line containing the highest amount of methyl salicylate. These glycosylated volatiles are important hidden aroma resources, and careful consideration should be taken when processing fruits because they can release various odors into the derived products. The amounts of taste compounds and nutritional substances varied throughout each line; Kohama and Izumi Kugani-like could be distinguished from one another owing to their high sucrose content, whereas Ishikunibu had high citrate, aspartate, and choline contents. Additionally, the differentiation of all six cultivation lines was shown by the PCA of the combined data of free and glycosidically bound volatile and non-volatile components, demonstrating an overall statistical differentiation pattern. However, the discriminatory power of glycosidically bound volatiles did not considerably improve the separation patterns in the PCA score plots. This study provides insights into the volatile and non-volatile component profiles of Okinawan Shiikuwasha fruit from different cultivation lines, thus supplementing quality control, product development, and decision making in the citrus industry.

## Figures and Tables

**Table 1:** Free volatile components of Shiikuwasha fruit of different cultivation lines (µg/L).

RI DB5-MS	RI DB-Wax	Compound	Ishikunibu	Izumi Kugani-Like	Kaachi	Kohama	Nakamoto Seedless	Ogimi Kugani
		Monoterpene hydrocarbon						
927	-	*α*-Thujene	7.80 ± 1.33 c	4.02 ± 2.64 d	10.37 ± 2.29 b	1.27 ± 0.33 e	3.87 ± 0.77 d	13.16 ± 2.56 a
936	1027	*α*-Pinene	41.50 ± 11.51 b	10.58 ± 6.96 d	46.12 ± 5.51 a	2.54 ± 0.29 e	12.23 ± 0.69 d	35.68 ± 9.40 c
953	1066	Camphene	0.64 ± 0.40 a	0.13 ± 0.28 c	nd	nd	nd	0.41 ± 0.44 b
956	1212	Dehydrosabinene	1.15 ± 1.2 a	nd	nd	nd	nd	nd
981	1109	*β*-Pinene	49.99 ± 5.23 a	9.27 ± 6.62 e	45.23 ± 10.64 b	2.64 ± 2.28 f	11.47 ± 7.37 d	39.85 ± 4.69 c
989	1160	*β*-Myrcene	98.60 ± 51.77 b	7.57 ± 4.40 e	142.60 ± 16.39 a	4.31 ± 4.18 e	17.55 ± 7.08 d	36.35 ± 0.55 c
1009	1165	*α*-Phellandrene	10.44 ± 4.40 a	0.92 ± 0.81 c	10.05 ± 2.33 a	nd	2.49 ± 2.44 b	3.16 ± 2.77 b
1027	1273	*p*-Cymene	976.78 ± 144.39 b	50.12 ± 24.49 e	242.01 ± 15.84 c	59.05 ± 22.52 de	76.82 ± 26.66 d	575.60 ± 99.45 b
1032	1202	Limonene	4371.23 ± 880.51 b	520.56 ± 276.18 e	5649.59 ± 888.97 a	251.39 ± 96.65 f	1310.56 ± 307.08 d	1614.14 ± 367.51 c
1045	-	*β*-Ocimene	7.60 ± 2.98 b	2.68 ± 0.74 d	15.35 ± 6.01 a	0.71 ± 1.28 e	1.17 ± 1.33 e	6.64 ± 2.67 c
1060	1247	*γ*-Terpinene	1441.03 ± 265.75 b	115.29 ± 50.82 e	1559.85 ± 242.92 a	23.56 ± 4.83 f	302.63 ± 49.09 d	665.42 ± 144.57 c
1088	1286	Terpinolene	74.56 ± 20.27 a	6.10 ± 2.59 e	70.52 ± 8.68 b	1.18 ± 0.60 f	17.17 ± 4.69 d	24.76 ± 17.00 c
1092	-	*β*-Methylisoallylbenzene	18.80 ± 13.96 a	1.15 ± 3.75 d	6.83 ± 16.20 b	0.56 ± 0.90 d	2.43 ± 4.54 cd	3.92 ± 8.48 c
1115	1252	*p*-Mentha-1,3,8-triene	2.77 ± 1.26 a	0.05 ± 0.10 d	0.49 ± 1.18 b	nd	0.14 ± 0.17 cd	0.29 ± 0.53 c
1138	-	*p*-Mentha-1,5,8-triene	3.58 ± 1.45 a	0.69 ± 0.20 b	0.46 ± 0.53 c	nd	0.07 ± 0.14 d	0.28 ± 0.54 c
		Monoterpene alcohol						
1099	1550	Linalool	345.20 ± 66.38 b	1.12 ± 0.54 d	423.90 ± 125.03 a	3.29 ± 0.98 d	27.57 ± 3.78 c	8.25 ± 5.25 d
1182	1608	Terpinen-4-ol	126.65 ± 29.71 a	1.06 ± 0.27 e	63.80 ± 14.85 b	0.98 ± 0.23 e	11.00 ± 2.65 d	19.42 ± 4.72 c
1188	-	*p*-Cymenol	3.47 ± 4.85 a	0.34 ± 0.09 c	0.64 ± 3.46 c	nd	1.82 ± 0.38 b	0.36 ± 0.35 c
1197	1702	*α*-Terpineol	93.31 ± 27.87 a	0.65 ± 1.12 d	37.25 ± 0.35 b	nd	8.07 ± 1.62 c	8.22 ± 2.89 c
1220	-	(*E*)-Carveol	2.24 ± 0.67	nd	nd	nd	nd	nd
1225	-	Citronellol	5.66 ± 7.30	nd	nd	nd	nd	nd
		Monoterpene ether						
1035	-	1,8-Cineole	nd	nd	nd	13.36 ± 11.90 a	nd	nd
		Monoterpene ketone						
1151	-	Camphor	4.21 ± 1.75 a	nd	2.54 ± 0.75 b	nd	0.70 ± 0.35 c	0.30 ± 0.11 d
1246	-	Carvone	6.11 ± 3.14 a	nd	nd	nd	nd	nd
		Sesquiterpene hydrocarbon						
1381	-	Copaene	nd	nd	1.82 ± 0.23 a	nd	nd	0.73 ± 0.28 b
1429	-	Caryophyllene	nd	nd	1.25 ± 0.44 a	nd	nd	2.32 ± 0.46 b
1440	-	*α*-Bergamotene	nd	nd	3.15 ± 1.18 b	nd	nd	nd
1505	-	*α*-Farnesene	6.30 ± 6.73 a	nd	nd	nd	1.65 ± 1.59 b	nd
1524	-	*δ*-Cadinene	nd	nd	1.57 ± 1.26 a	nd	0.55 ± 0.22 b	0.69 ± 0.16 c
		Alcohol						
1068	-	1-Octanol	11.91 ± 32.15 a	nd	11.64 ± 31.10 a	nd	nd	0.61 ± 0.73 b
		Aldehyde						
800	1082	Hexanal	14.62 ± 9.15 d	8.38 ± 13.06 e	35.55 ± 2.93 c	82.35 ± 46.08 a	nd	69.36 ± 11.49 b
901	1187	Heptanal	1.53 ± 3.68 bc	0.53 ± 1.19 d	0.95 ± 1.52 bcd	2.99 ± 4.89 a	0.88 ± 2.25 cd	1.65 ± 0.54 b
1003	1291	Octanal	4.57 ± 2.22 b	0.45 ± 0.99 f	5.73 ± 0.77 a	4.00 ± 3.51 c	2.16 ± 1.62 d	1.63 ± 0.62 e
1104	1400	Nonanal	5.92 ± 4.24 a	0.69 ± 0.32 b	4.32 ± 14.48 a	2.17 ± 0.67 b	1.69 ± 3.08 b	2.17 ± 0.66 b
1206	-	Decanal	3.06 ± 5.55 b	nd	4.44 ± 1.90 a	0.19 ± 0.11 c	0.43 ± 0.39 c	nd
		Ester						
1208	-	Octyl acetate	1.38 ± 5.12 b	nd	3.12 ± 3.55 a	nd	nd	nd
		Ether						
1229	1442	Methylthymol	1.86 ± 2.05 a	1.63 ± 1.29 a	nd	nd	nd	nd

Values are presented as the mean ± standard deviation of three replicates; nd = not detected. Means in the same row followed by the same letter are not significantly different (*p* < 0.05). Analysis on the DB5-MS column was used for value quantification.

**Table 2:** Odor activity values (OAVs) of aroma-active compounds of Shiikuwasha fruit of different cultivation lines.

Compound	Odor Threshold (µg/L)	Ishikunibu	Izumi Kugani-like	Kaachi	Kohama	Nakamoto Seedless	Ogimi Kugani
*β*-Myrcene	16.6	5.94	0.46	8.59	0.26	1.06	2.19
*p*-Cymene	13.3	73.44	3.77	18.20	4.44	5.78	43.28
Limonene	34	128.57	15.31	166.16	7.39	38.55	47.47
Linalool	1.5	230.13	0.75	282.60	2.19	18.38	5.50
1,8-Cineole	0.26	nd	nd	nd	51.39	nd	nd
Caryophyllene	160	nd	nd	0.01	nd	nd	1.45
Octanal	0.1	45.72	4.53	57.28	39.98	21.56	16.29
Nonanal	3.5	1.69	0.20	1.23	0.62	0.48	0.62

Odor threshold concentration of the compound [19]. The OAV was calculated by dividing the compound concentration by its odor threshold; nd: not detected. Compounds with an OAV greater than one were considered as aroma-active compounds.

**Table 3:** Released compounds from glycosidically bound volatile components of Shiikuwasha fruit of different cultivation lines (µg/L).

RI DB5-MS	RI DB-Wax	Compound	Ishikunibu	Izumi Kugani-like	Kaachi	Kohama	Nakamoto Seedless	Ogimi Kugani
		Aliphatic alcohol						
747	1209	3-Methyl-1-butanol	11.41 ± 0.75 d	48.66 ± 9.72 a	26.83 ± 5.40 bc	9.28 ± 1.84 d	12.17 ± 5.52 cd	29.89 ± 4.67 b
750	-	2-Methyl-1-butanol	3.25 ± 0.59 b	20.41 ± 3.77 ab	7.01 ± 2.42 b	6.39 ± 1.61 b	28.55 ± 15.90 a	6.53 ± 2.07 b
775	1251	3-Methyl-2-butenol	6.59 ± 1.24 b	19.97 ± 3.57 a	3.56 ± 0.94 bc	nd	6.73 ± 3.49 b	nd
769	1331	1-Pentanol	1.61 ± 0.71 c	3.78 ± 0.62 c	37.54 ± 5.53 a	19.63 ± 1.41 b	11.38 ± 2.64 bc	34.62 ± 7.85 a
839	1318	3-Methyl-1-pentanol	27.22 ± 2.93 a	33.56 ± 8.89 a	8.31 ± 0.41 b	11.43 ± 3.39 b	13.58 ± 4.05 b	12.48 ± 3.01 b
849	1391	3-*(Z)*-Hexenol	14.88 ± 0.70 b	30.86 ± 3.87 a	5.25 ± 0.71 cd	nd	nd	6.09 ± 3.24 c
862	1358	1-Hexanol	8.25 ± 0.51 c	22.19 ± 18.23 c	284.01 ± 368.54 a	21.76 ± 50.08 c	11.70 ± 38.00 c	212.62 ± 243.53 b
898	1324	2-Heptanol	nd	nd	1.41 ± 1.36 b	nd	nd	5.98 ± 2.94 a
977	1454	1-Octen-3-ol	nd	nd	18.04 ± 5.32 b	0.93 ± 0.32 c	nd	27.92 ± 6.12 a
995	1400	3-Octanol	nd	nd	3.90 ± 2.04 a	nd	nd	8.46 ± 7.48 a
1000	1425	2-Octanol	nd	nd	9.24 ± 5.25	nd	nd	nd
1068	1883	1-Octanol	25.38 ± 2.78 b	nd	50.34 ± 5.04 a	nd	nd	nd
		Aromatic alcohol						
1033	2006	Benzyl alcohol	61.60 ± 10.25 b	83.38 ± 20.96 ab	70.20 ± 12.12 b	26.79 ± 2.12 b	57.51 ± 6.57 b	145.29 ± 53.32 a
1048	1562	2-Methylphenol	nd	7.76 ± 2.05 ab	3.85 ± 0.28 ab	1.61 ± 0.85 ab	3.16 ± 0.63 ab	10.42 ± 8.49 a
1112	1477	Phenylethyl alcohol	14.05 ± 2.89 a	3.85 ± 1.87 bc	1.82 ± 0.42 c	nd	5.10 ± 0.87 bc	10.00 ± 5.02 ab
1194	1450	Methyl salicylate	3.54 ± 1.13 b	68.83 ± 22.67 a	3.24 ± 1.15 b	1.50 ± 0.87 b	5.44 ± 7.19 b	19.75 ± 11.54 b
		Monoterpene alcohol						
1072	1919	(*Z*)-Linalool oxide	nd	nd	8.68 ± 5.58 ab	nd	1.31 ± 0.88 bc	10.00 ± 3.70 a
1087	1781	(*E*)-Linalool oxide	nd	0.64 ± 1.11 ab	nd	1.81 ± 0.55 a	1.15 ± 0.26 ab	nd
1288	2200	Thymol	nd	17.65 ± 6.92 a	nd	2.76 ± 0.56 b	nd	nd
1352	2176	Eugenol	2.31 ± 0.74 bc	nd	4.42 ± 2.11 ab	nd	6.29 ± 0.63 a	4.28 ± 0.65 ab

Values are presented as the mean ± standard deviation of three replicates; nd = not detected. Means in the same row followed by the same letter are not significantly different (*p* < 0.05). Analysis on the DB5-MS column was used for value quantification.

**Table 4:** Non-volatile components of Shiikuwasha fruit of different cultivation lines (mM).

Compound	Ishikunibu	Izumi Kugani-like	Kaachi	Kohama	Nakamoto Seedless	Ogimi Kugani
Sugar						
Sucrose	26.08 ± 1.32 c	33.38 ± 0.19 ab	26.99 ± 2.12 c	36.21 ± 3.27 a	23.87 ± 0.89 c	28.55 ± 2.17 bc
Fructose	26.22 ± 1.32 bc	22.14 ± 0.56 c	31.25 ± 1.96 a	25.36 ± 1.16 bc	31.13 ± 2.84 a	28.92 ± 0.76 ab
Glucose	26.91 ± 1.66 b	20.75 ± 0.16 c	28.95 ± 1.49 ab	23.71 ± 0.49 c	31.02 ± 1.32 a	22.38 ± 0.26 c
Organic acid						
Citrate	42.04 ± 0.51 a	5.60 ± 0.08 e	21.85 ± 1.16 b	16.68 ± 0.46 c	5.66 ± 0.28 e	12.85 ± 0.33 d
Malate	3.66 ± 0.41 b	1.92 ± 0.02 c	4.80 ± 0.33 a	4.72 ± 0.38 a	3.65 ± 0.32 b	4.78 ± 0.36 a
Malonate	0.25 ± 0.02 d	0.38 ± 0.01 c	0.53 ± 0.04 b	0.58 ± 0.01 b	0.65 ± 0.01 a	0.42 ± 0.02 c
Aspartate	2.04 ± 0.22 a	0.25 ± 0.01 e	1.27 ± 0.07 b	0.54 ± 0.01 d	0.52 ± 0.02 de	1.00 ± 0.02 c
Amino acid						
GABA	0.85 ± 0.01 a	0.48 ± 0.04 c	0.79 ± 0.05 a	0.59 ± 0.02 b	0.87 ± 0.04 a	0.67 ± 0.06 b
Vitamin						
Choline	0.15 ± 0.01 a	0.08 ± 0.01 c	0.08 ± 0.00 c	0.07 ± 0.00 c	0.13 ± 0.01 b	0.09 ± 0.01 c

Values are presented as the mean ± standard deviation of three replicates. Means in the same row followed by the same letter are not significantly different (*p* < 0.05).

## Data Availability

The original contributions presented in the study are included in the article and Supplementary Material, further inquiries can be directed to the corresponding author.

## Supplementary Materials

The following supporting information can be downloaded at: https://www.mdpi.com/article/10.3390/foods13213428/s1, Table S1: Morphological and physico-chemical characteristics of Shiikuwasha fruit of two biological replicates; Table S2: Variable importance in the projections (VIP) values of the combined normalized intensity data of free and glycosidically bound volatile and non-volatile components of Shiikuwasha fruit; Figure S1: (a) PLS-DA score plot, (b) PLS-DA factor loading, and (c) HCA plots of the combined normalized intensity data of free and glycosidically bound volatile and non-volatile components of Shiikuwasha fruit.
