# Supplementary material for: Characterization of Free and Glycosidically Bound Volatile and Non-Volatile Components of Shiikuwasha (Citrus depressa Hayata) Fruit
Source: Foods. 2024 Oct 28;13(21):3428. doi: 10.3390/foods13213428 (PMC11544857; doi:10.3390/foods13213428)
Supplement: Supplementary file 1 [file foods-13-03428-s001.zip › Supplementary Figure S1.pdf]

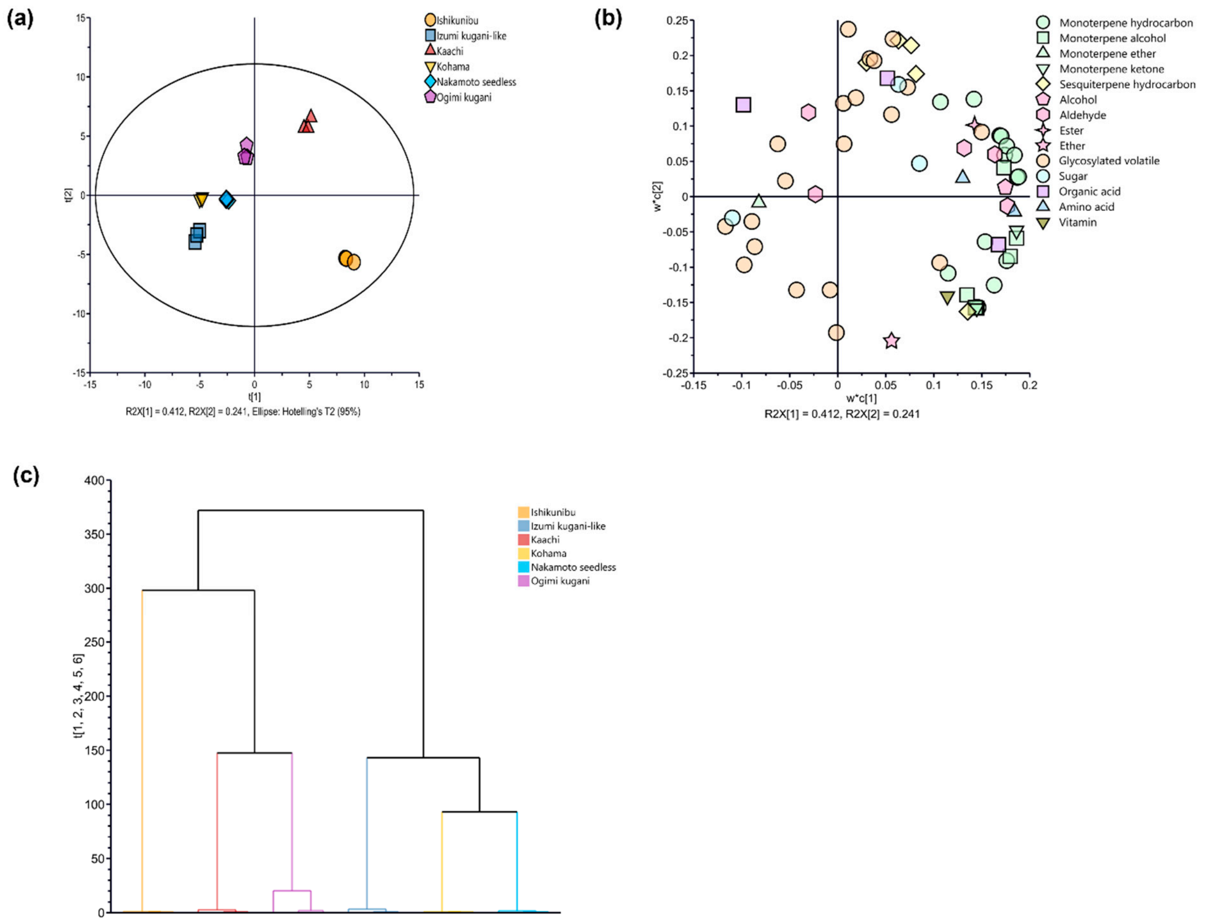

Supplementary Figure S1. (a) PLS-DA score plot, (b) PLS-DA factor loading, and (c) HCA plots of the combined normalized intensity data of free and glycosidically bound volatile and non-volatile components of Shiikuwasha fruit.
